# Supplementary material for: Charge and exciton dynamics of OLEDs under high voltage nanosecond pulse: towards injection lasing
Source: Nat Commun. 2020 Aug 27;11:4310. doi: 10.1038/s41467-020-18094-4 (PMC7453197; doi:10.1038/s41467-020-18094-4)
Supplement: Supplementary file 1 — Supplementary Information [file 41467_2020_18094_MOESM1_ESM.docx]

**Supplementary Information**

**Charge and exciton dynamics of OLEDs under high voltage nanosecond pulse: Towards injection lasing**

Viqar Ahmad^1,2,†^, Jan Sobus^1,2, †,^*^,^, Mitchell Greenberg^3,†^, Atul Shukla^1,2^, Bronson Philippa^3^, Almantas Pivrikas^4^, George Vamvounis^3^, Ronald White^3^, Shih-Chun Lo^2,5,^* & Ebinazar B. Namdas^1,2,^*

^1^School of Mathematics and Physics, University of Queensland, Brisbane, QLD 4072, Australia

^2^Centre for Organic Photonics & Electronics, University of Queensland, Brisbane, QLD 4072, Australia

^3^College of Science & Engineering, James Cook University, QLD 4811, Australia

^4^School of Engineering and Information Technology, Murdoch University, WA 6150, Australia

^5^School of Chemistry and Molecular Biosciences, University of Queensland, Brisbane, QLD 4072, Australia

**Index:**

**Supplementary Figure 1.** Structure of SY polymer and OLED with energy diagram.……………2

**Supplementary Figure 2.** OLED DC characteristics of SY devices.………………………..….…3

**Supplementary Figure 3.** Stability of SY OLEDs under high voltage pulse excitation.……….....4

**Supplementary Figure 4.** Current response of the SY OLED driven with 15 ns pulses.…….......5

**Supplementary Figure 5.** Simulated current response of the OLED – circuit model..…….…......6

**Supplementary Figure 6**. Results of LCR measurements of small area OLEDs.……………........7

**Supplementary Figure 7** Simulated temporal peak of exciton formation...………………..….…8

**Supplementary Figure 8**. Simulated and experimental OLED I-V characteristics..…………......9

**Supplementary Figure 9.** Drift-diffusion model to estimate singlet generation..……..………. .10

**Supplementary Figure 10**. Peak/averaged singlet concentrations shifting with current density...11

**Supplementary Figure 11**. Color bar time- space plots of singlet concentration…......................12

**Supplementary Figure 12**. Spatially averaged singlet concentrations for both models.…..........13

**Supplementary Figure 13**. Relative contributions of singlet decay pathways..………..………...14

**Supplementary Figure 14**. Pumping power density needed to generate singlets.….………........15

**Supplementary Figure 15**. Experimental and simulation results of F8BT and PFO OLEDs..….16

**Supplementary Figure 16.** I-V and current efficiency characteristics of OLEDs........................ 17

**Supplementary Figure 17.** Relative exciton decay pathways in SY, F8BT and PFO OLEDs..... 18

**Supplementary Figure 18**. Simulation results of lasing thresholds for F8BT and PFO..…………19

**Supplementary Figure 19.** Simulated singlet densities in BSBCz devices……………………...20

**Supplementary Figure 20.** Relative magnitudes of light outcoupling channels.….……………...21

**Supplementary Figure 21.** Pumping thresholds of champion lasing materials………………….22

**Supplementary Figure 22.** Structures of hole-only (HOD) and electron-only (EOD) devices….23

**Supplementary Figure 23.** Results of field induced quenching PL study..…..…………….........24

**Supplementary Figure 24.** Measurement of singlet-singlet annihilation (SSA) constant (*k_ss_*).......25

**Supplementary Figure 25.** Schematic diagram of high voltage pulse measurement setup...........26

**Supplementary Table 1.** Values of contact resistance R_s_ and layer resistance R_layer_ …………....27

**Supplementary Table 2**. Decay and annihilation rates of F8BT..……………….…….………...28

**Supplementary Table 3**. Decay and annihilation rates of PFO……………………….….……...29

**Supplementary Table 4**. Decay and annihilation rates of BSBCz………………………………30

**Supplementary Table 5.** Best performing small molecules and oligomers...................................31

**Supplementary Table 6.** Best performing dendrimers and polymers............................................32

**Supplementary Table 7**. Estimation of singlet-singlet annihilation rate…………………...........33

**Supplementary Note 1.** Circuit model – square pulse response ………………………………….34

**Supplementary Note 2.** Circuit model – trapezoid pulse response ……………….….………….35

**Supplementary Note 3.** Drift-diffusion model………………………..……………….………...36

**Supplementary Note 4.** Hole and electron-only devices and field quenching experiments……..39

**Supplementary Note 5.** Measurement of singlet-singlet annihilation (SSA) constant (*k_ss_***)**……...40

**Supplementary Note 6** Light outcoupling………………………………………………………..41

**Supplementary References**.……………………………………………………………...……...42


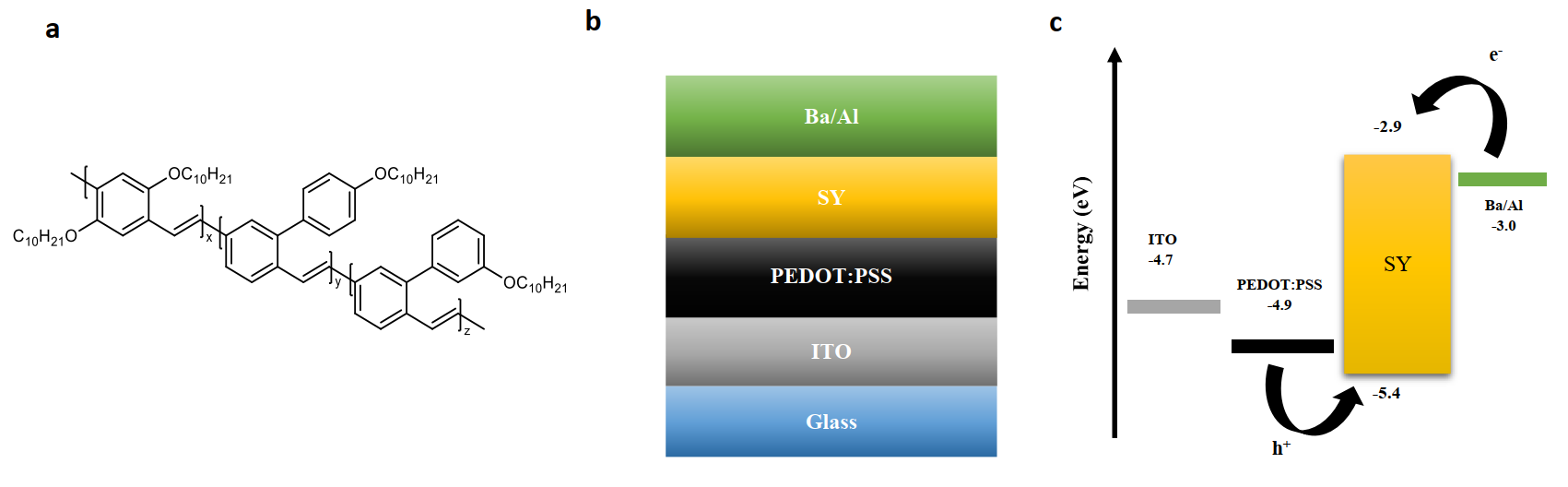


**Supplementary Figure 1. Structure of SY polymer and OLED with energy diagram.
a** Molecular structure of Super Yellow (SY), **b** OLED device structure on a 0.55 mm thick glass, **c** Energy level diagram of the OLED with SY as the emitter.


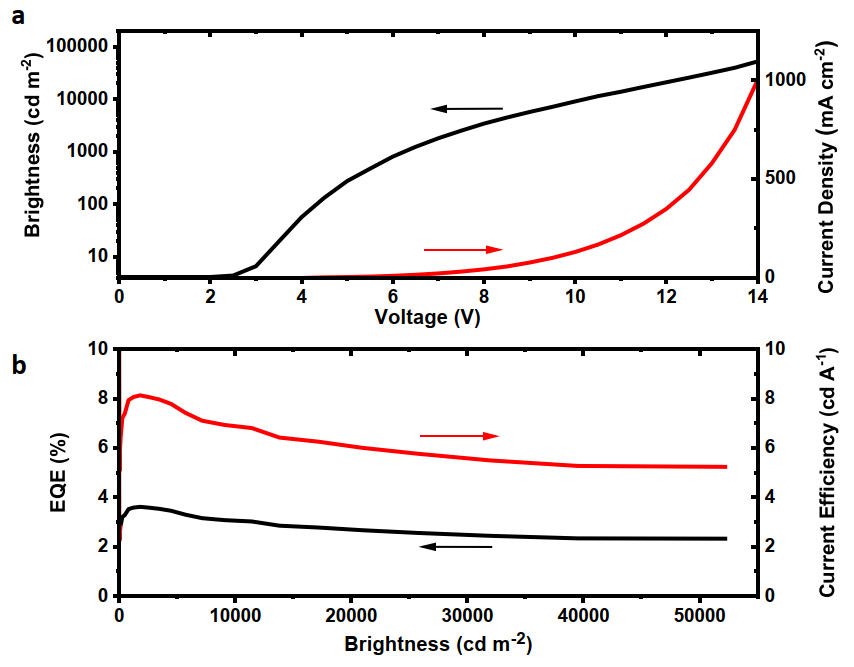


**Supplementary Figure 2. OLED DC characteristics of SY devices.** **a** J-V-L curve, **b** EQE and current efficiency of the device.


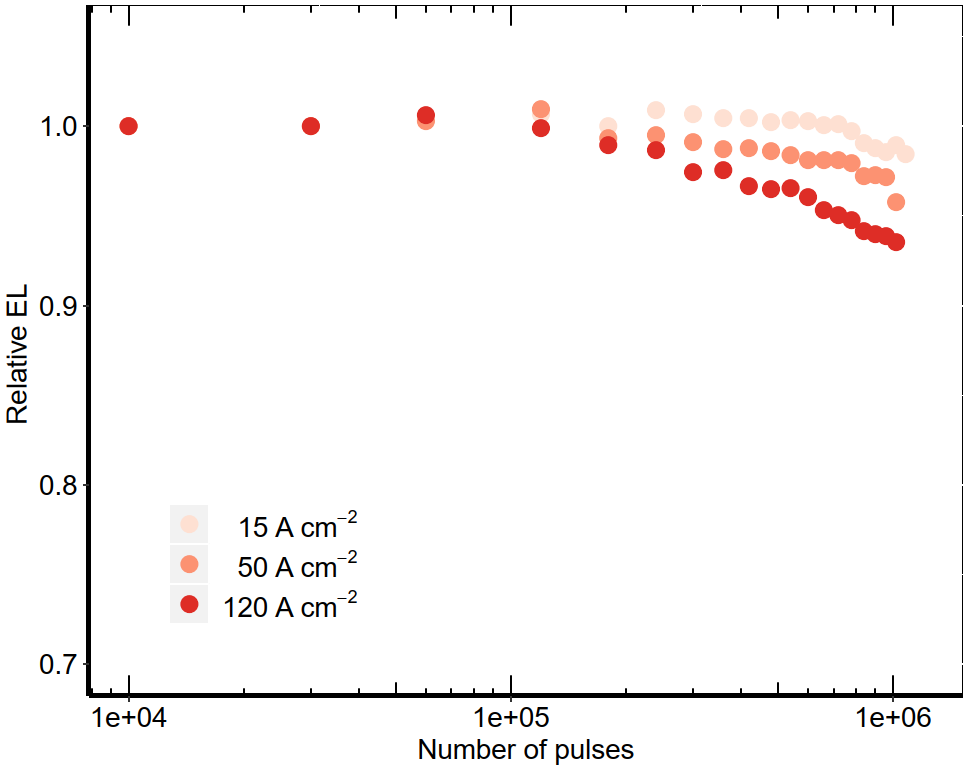


**Supplementary Figure 3. Stability of SY OLEDs under high voltage pulse excitation.** Relative electroluminescence of the SY based OLEDs as a function of the number of applied high voltage pulses (pulse width is 300 ns). Over 90% of the signal remains even after one million pulses at 120 A cm^-2^.


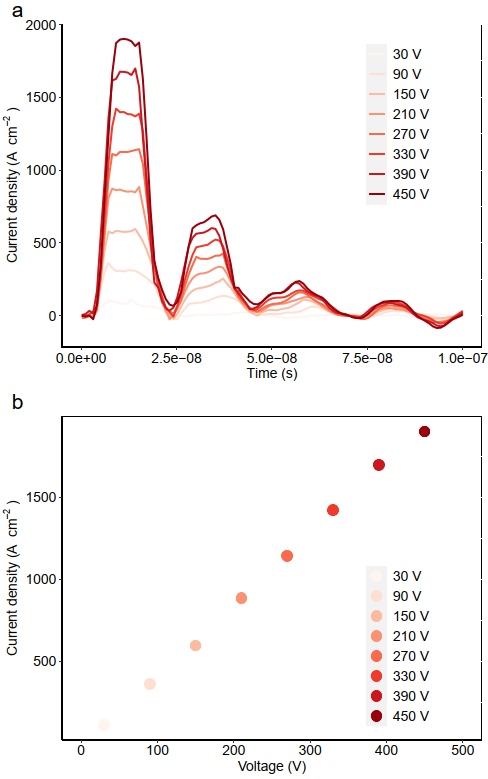


**Supplementary Figure 4. Current response of the SY OLED driven with 15 ns pulses.** The voltages ranged from 30 V to 450 V. **a** Transient current density response, having ringing effects due to short pulses. **b** Plot of the peak current density versus applied voltage for this device.


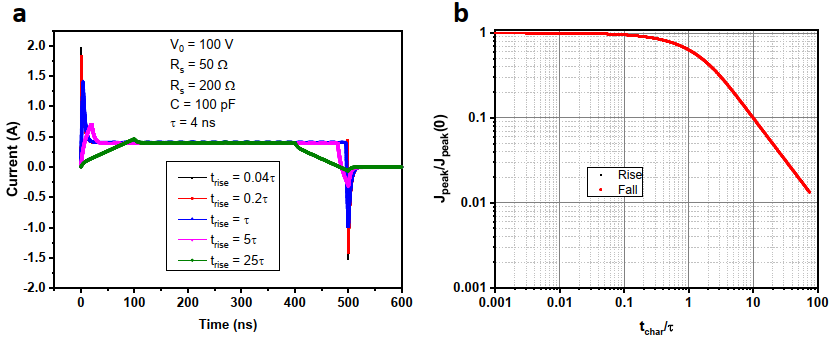


**Supplementary Figure 5. Simulated current response of the OLED – circuit model. a** Under electrical excitation with varying rise/fall times of the input voltage pulse (in reference to characteristic time of the device), relative amplitudes of the current peaks. **b** Relation of normalised peak current intensity in relation to rise/fall times of the input voltage.


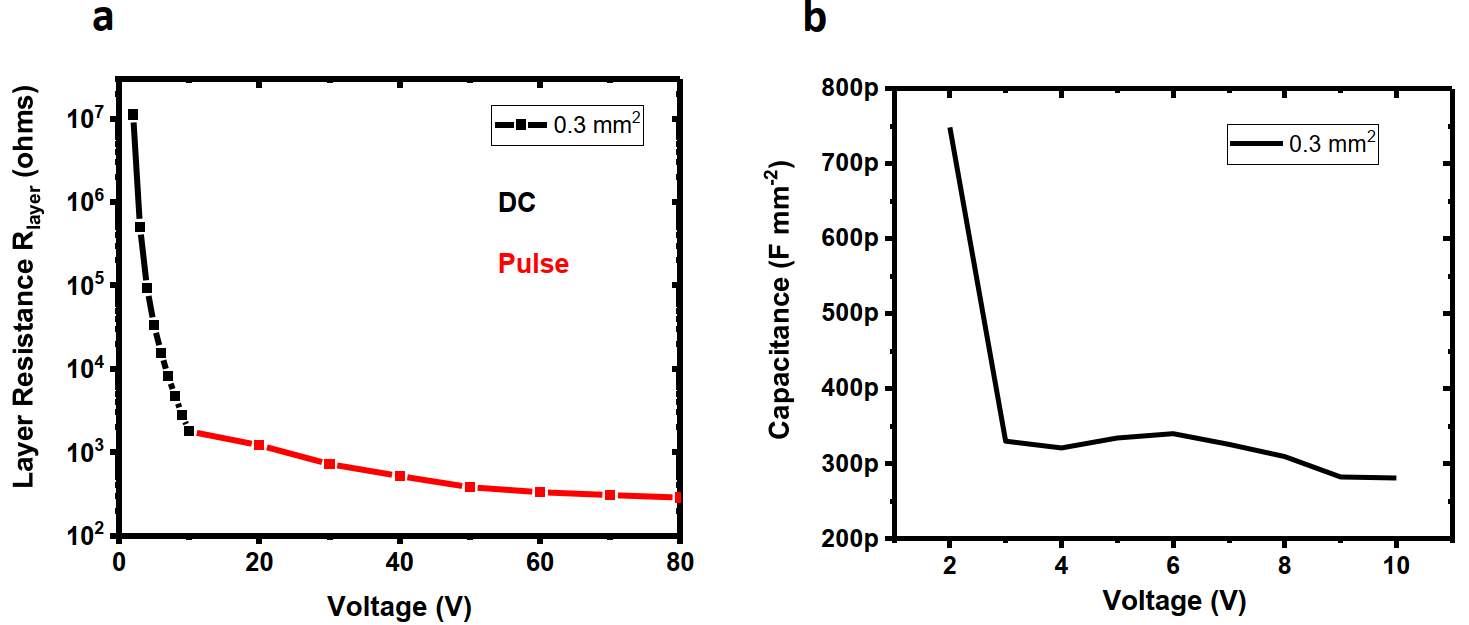


**Supplementary Figure 6.** **Results of LCR measurements of small area OLEDs. a** Layer resistance as a function of DC voltage for a 0.3 mm^2^ OLED is shown. As voltage increases, charges get pushed into the organic layer, lowering layer resistance. Black dots represent layer resistance extracted from LCR (DC) measurement and red dots represent layer resistance extracted from high voltage pulse measurements. **b** Organic layer capacitance as a function of voltage, the huge spike at 2 V is due to charge accumulation at the organic layer interface prior to turn on which steadies to a lower value after turn on.


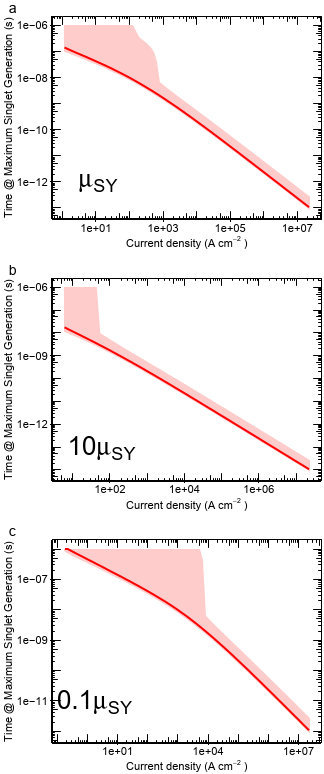


**Supplementary Figure 7. Simulated temporal peak of exciton formation.** It is plotted as a function of injected current density and mobility of the emissive layer**.** **a** At 1/10^th^ SY mobility. **b** At SY mobility. **c** At 10 times SY mobility. Red shade indicates time interval where the recombination rate is at least 50% of the maximum value for given current density.


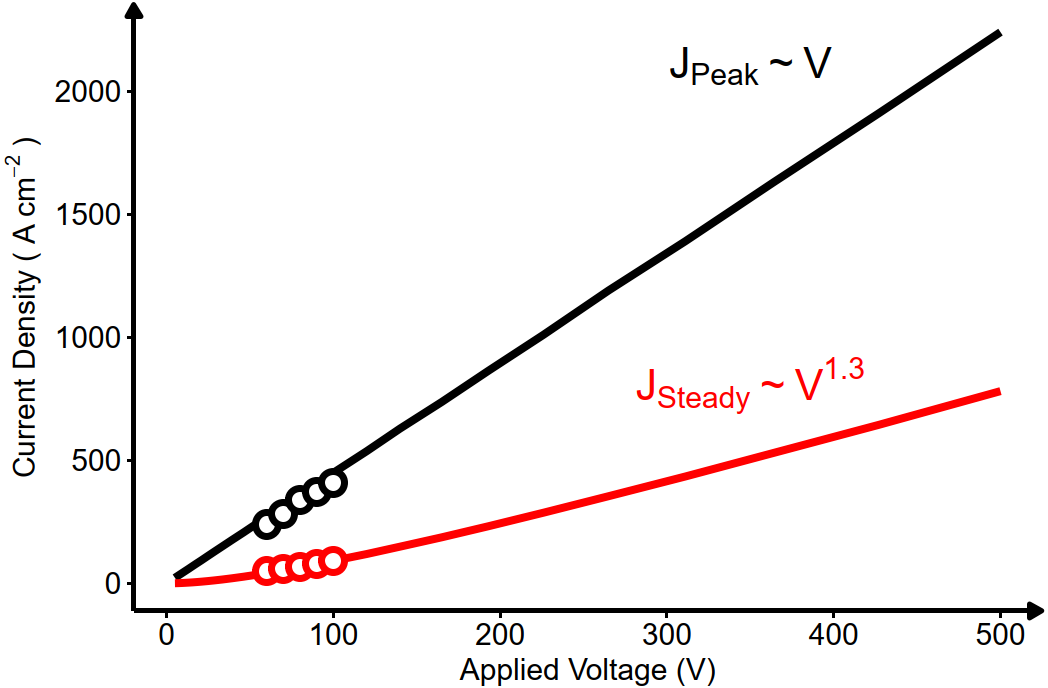


**Supplementary Figure 8.** **Simulated and experimental OLED I-V characteristics.** Simulated (lines) and experimental (points) current densities of SY OLEDs under pulsed excitation. Peak and steady state values were fitted against voltage using power dependence. Obtained power law is included near the corresponding curves.


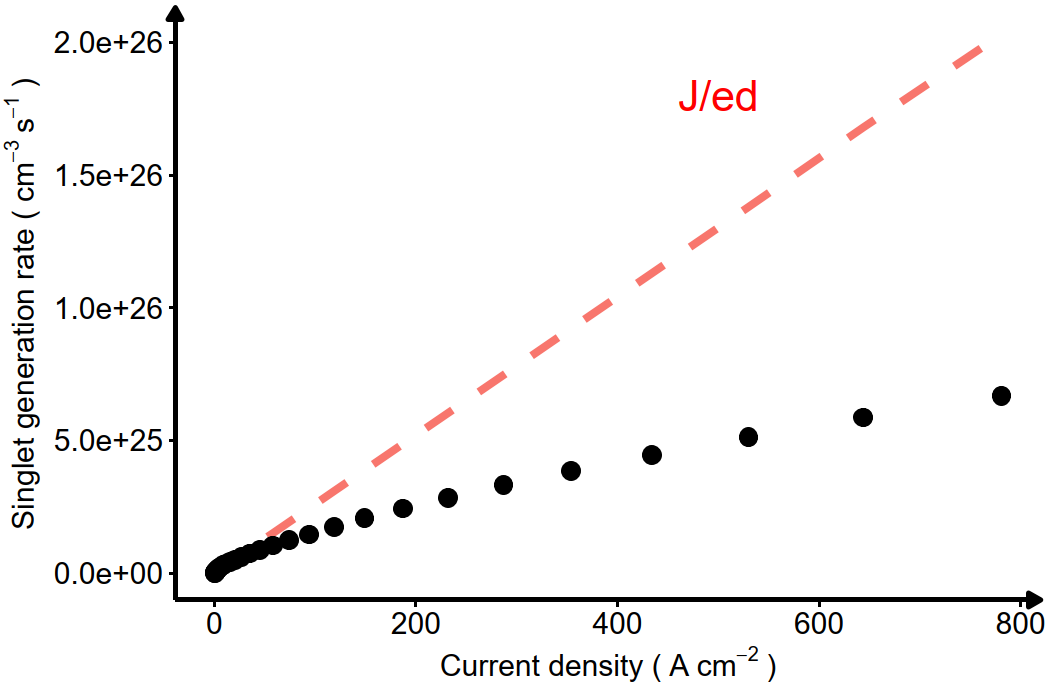


**Supplementary Figure 9.** **Drift-diffusion model estimate of singlet generation.** Maximum singlet exciton generation rate extracted from the drift-diffusion model (black points) with commonly used J/ed approximation as a reference.


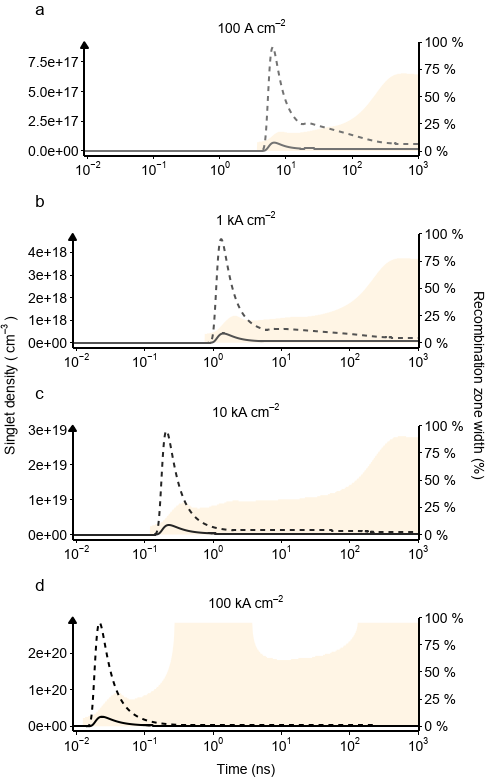


**Supplementary Figure 10.** **Peak/averaged singlet concentrations shifting with current density.** Maximum (dashed) and averaged over the recombination zone (solid) singlet concentrations for different current densities. Orange areas indicate width of the recombination zone as a fraction of total organic layer thickness. **a** 100 A cm^-2^. **b** 1,000 A cm^-2^. **c** 10,000 A cm^-2^. **d** 100,000 A cm^-2^.


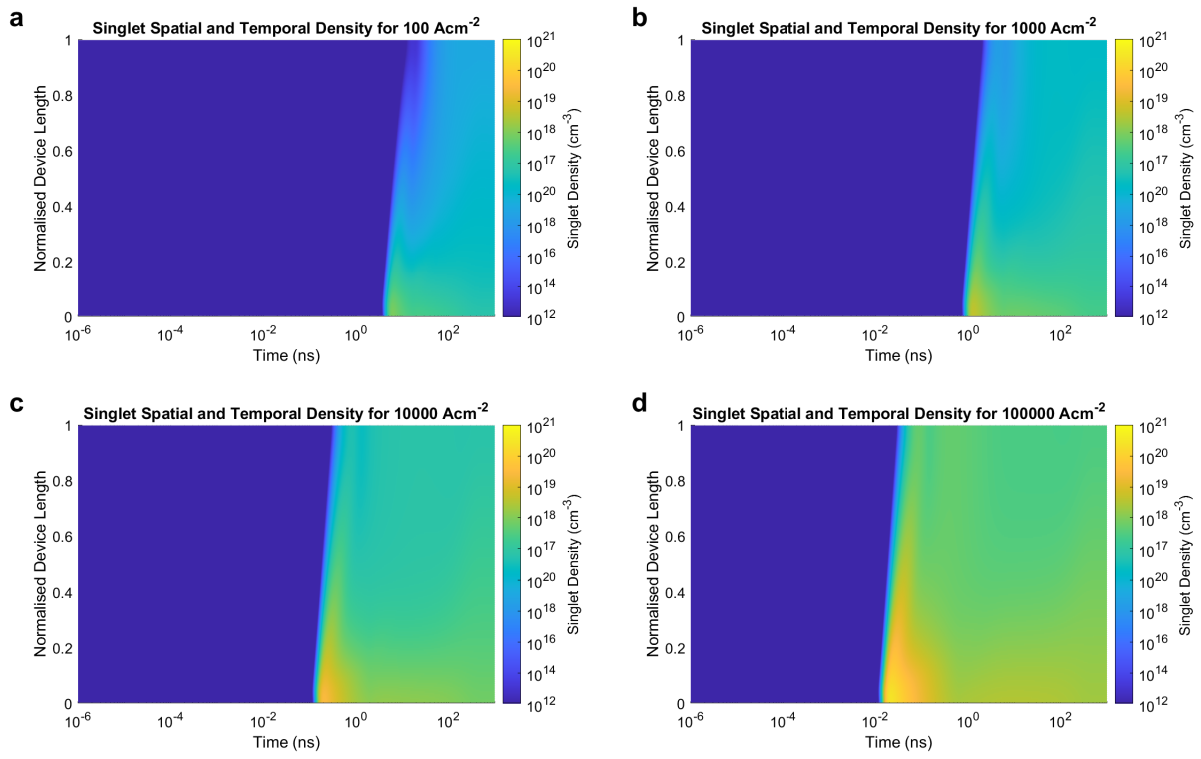


**Supplementary Figure 11. Colorbar time-space plots of singlet concentrations.** Singlet densities are mapped as a function of time and position across the organic layer when subject to pumping at different current densities. **a** 100 A cm^-2^. **b** 1,000 A cm^-2^. **c** 10,000 A cm^-2^. **d** 100,000 A cm^-2^.


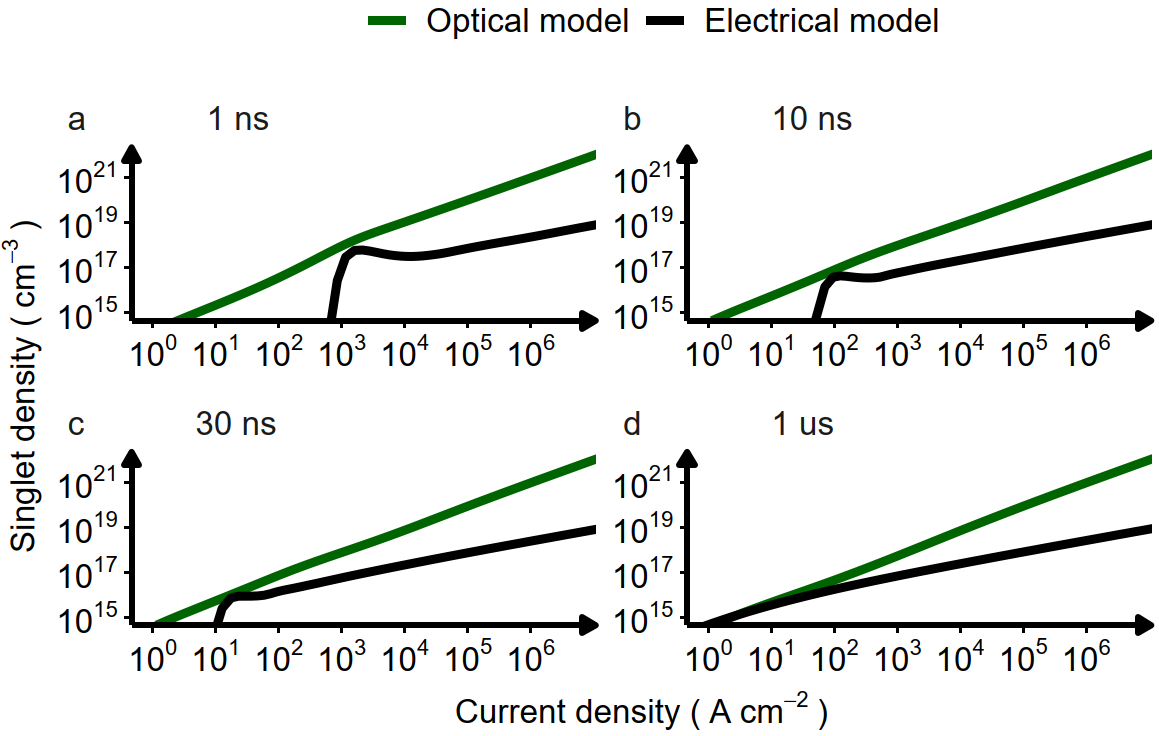


**Supplementary Figure 12.** **Spatially averaged singlet concentrations for both models.** They are plotted as a function of current density, at different times after the pulse onset. **a** 1 ns. **b** 10 ns. **c** 30 ns. **d** 1 µs.


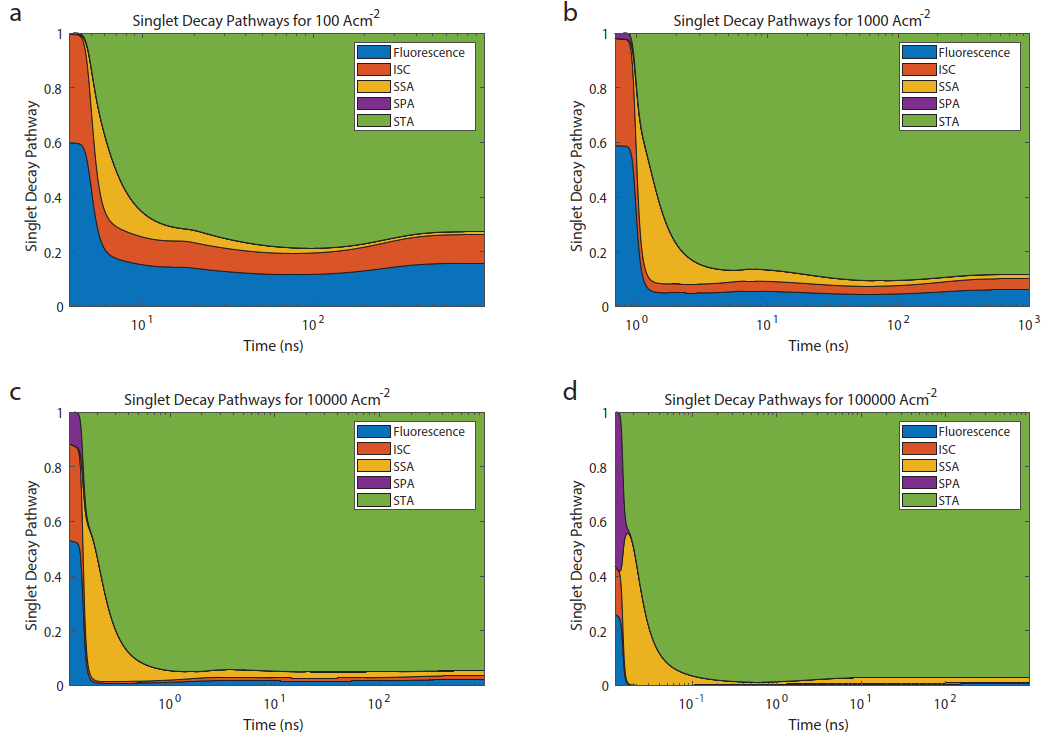


**Supplementary Figure 13.** **Relative contributions of singlet decay pathways.** Here, ISC, SSA, SPA and STA stand for intersystem crossing, singlet-singlet annihilation, singlet-polaron-annihilation and singlet-triplet annihilation respectively. They are plotted for different densities of injection current **a** 100 A cm^-2^. **b** 1,000 A cm^-2^. **c** 10,000 A cm^-2^. **d** 100,000 A cm^-2^.


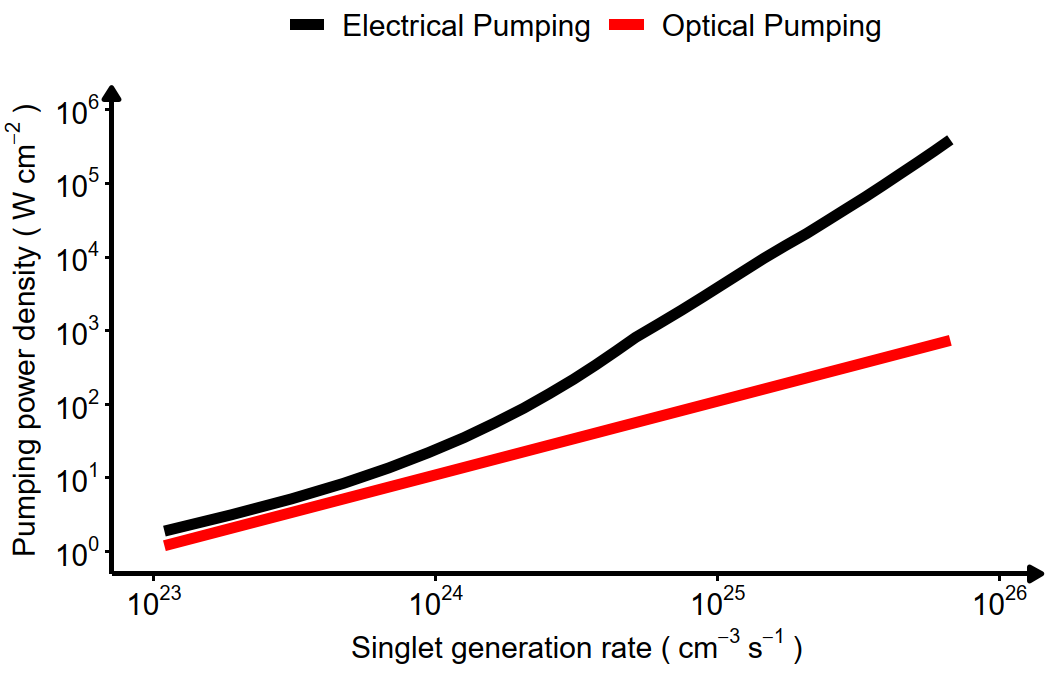


**Supplementary Figure 14.** **Pumping power density needed to generate singlets.** For singlet generation rates of 10^24^ (corresponding to steady singlet concentration of around 10^15^) or higher, the power density required to maintain singlet generation can be orders of magnitude higher in electrical mode than in optical mode raising power dissipation issues.


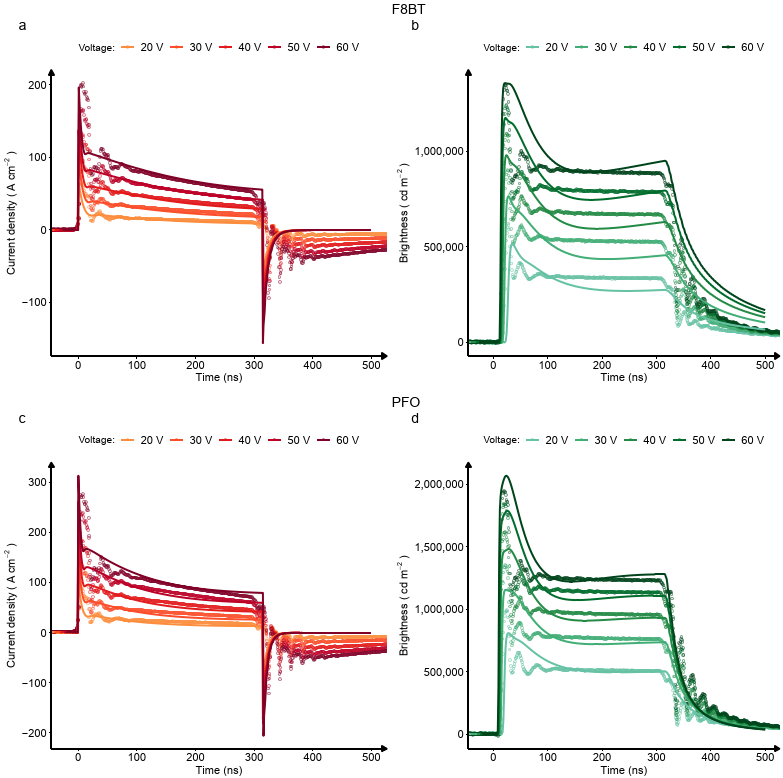


**Supplementary Figure 15. Experimental and simulation results** **of F8BT** **and PFO** **OLEDs.
a** Experimental and fitted current density response of F8BT. **b** Experimental and fitted EL response of F8BT. **c** Experimental and fitted current density response of PFO. **d** Experimental and fitted EL response of PFO. Optical and electrical lasing threshold calculation for current/singlet densities and respective peak singlet location in ns.


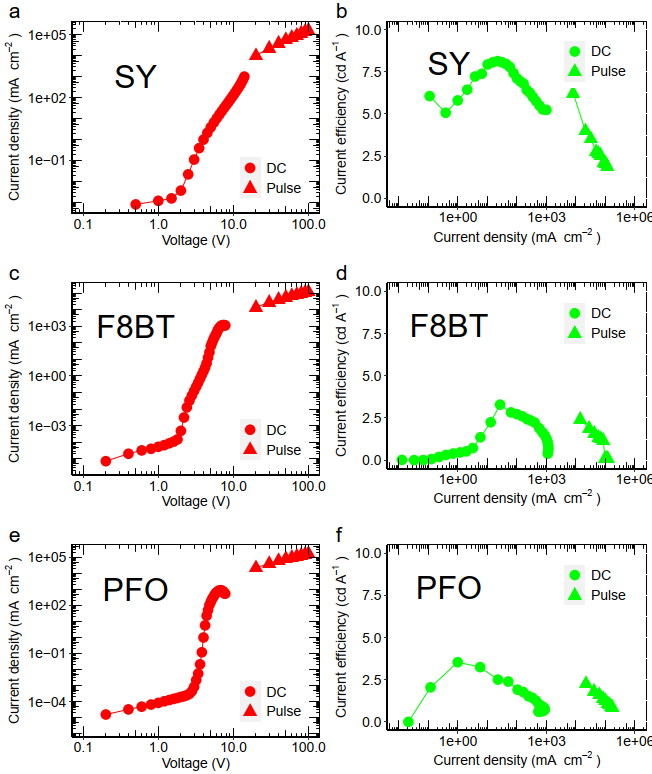


**Supplementary Figure 16. I-V and current efficiency characteristics of OLEDs.** Both DC and pulse regimes are included with current densities plotted in red and current efficiencies in green
**(a**, **b)** for SY, **(c**, **d)** for F8BT, and **(e**, **f)** for PFO.


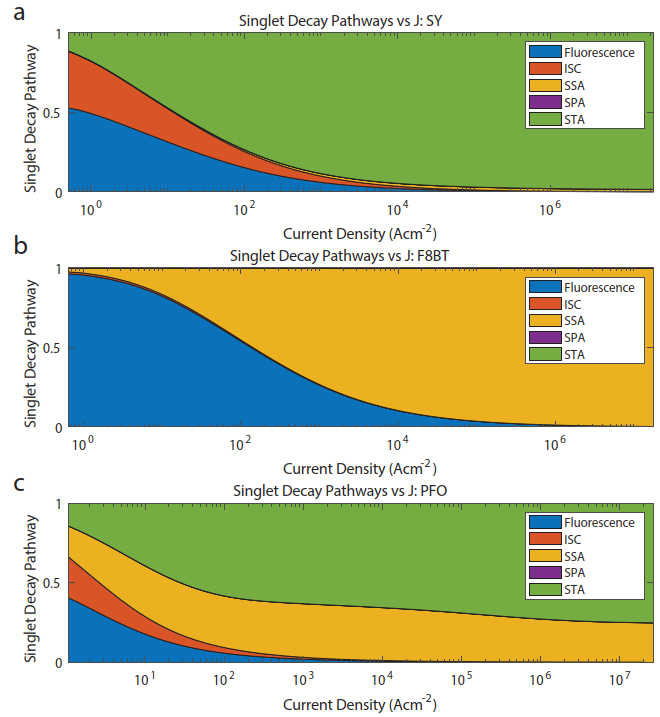


**Supplementary Figure 17** **Relative exciton decay pathways in SY, F8BT and PFO OLEDs.** Here they are plotted as a function of pumping current density. Main processes responsible for EQE roll-off are **a** STA in the case of SY. **b** SSA for F8BT. **c** A mix of both for PFO. Unsurprisingly, bimolecular processes are dominant at higher current densities.


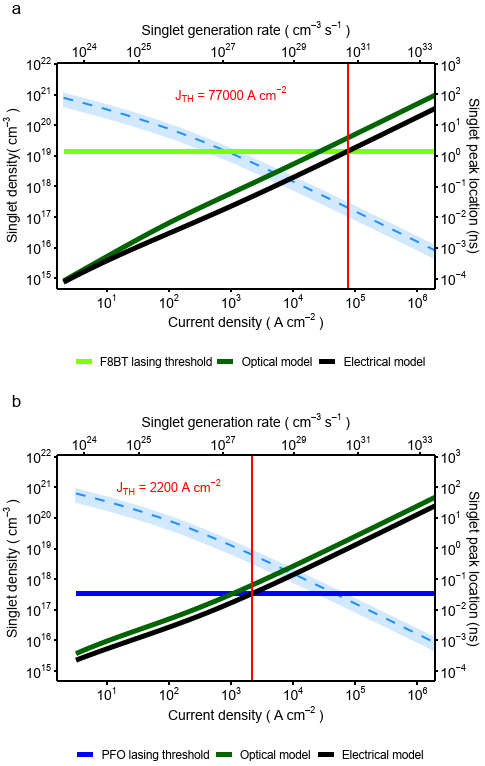


**Supplementary Figure 18. Simulation results** **of lasing thresholds for F8BT and PFO**
**a** Optical and electrical lasing threshold calculation for current/singlet densities and respective peak singlet location in ns for F8BT. **b** optical and electrical lasing threshold calculation for current/singlet densities and respective peak singlet location in ns for PFO.


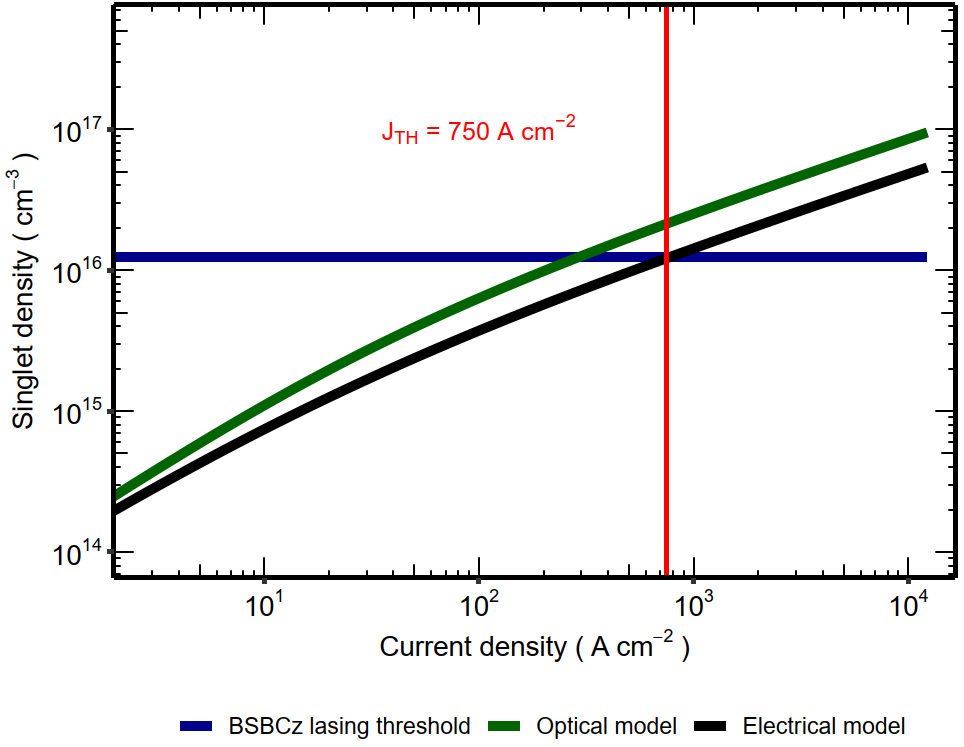


**Supplementary Figure 19.** **Simulated singlet densities in BSBCz devices** reported by C. Adachi^14^ subject to optical and electrical pumping and obtained threshold current density.


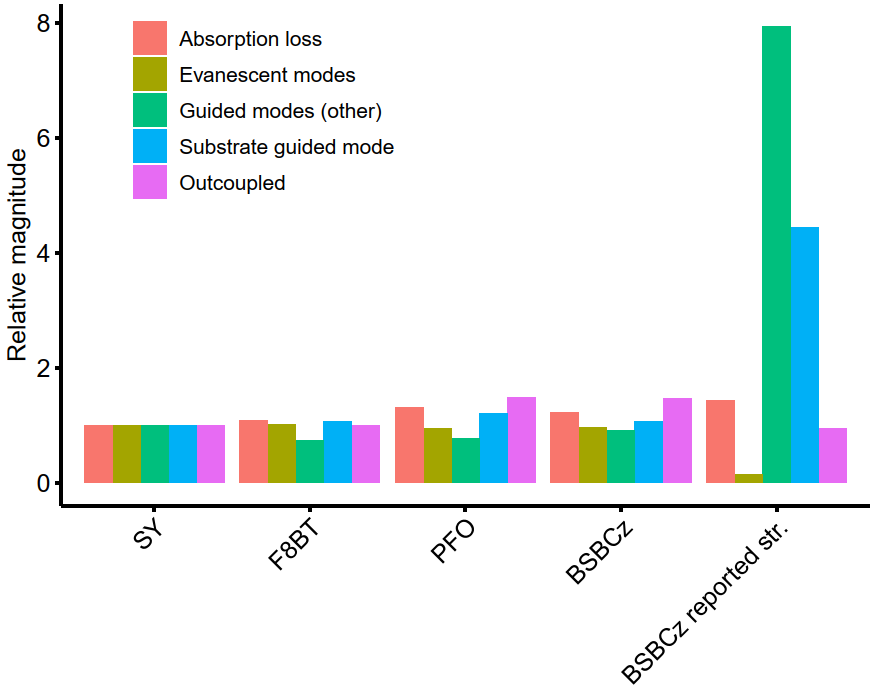


**Supplementary Figure 20. Relative magnitudes of light outcoupling channels.** SY based device is taken as reference. Results are shown for same architecture devices employing other emitters (polymer and small molecule) and BSBCz device of the structure reported by Adachi’s group^14^.


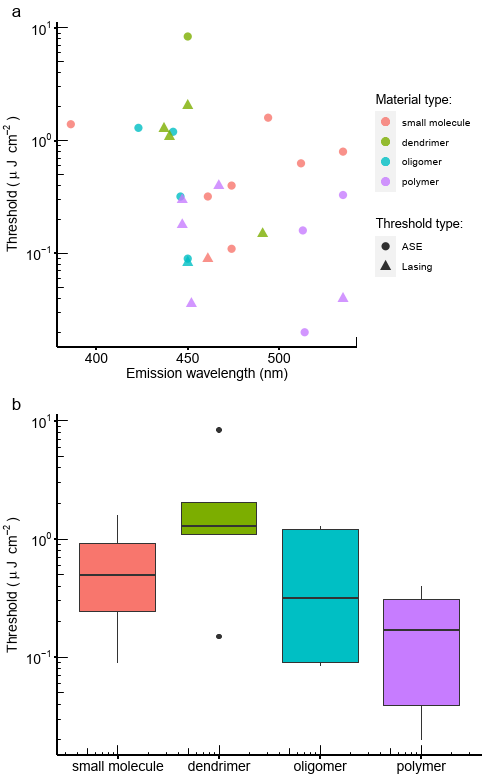


**Supplementary Figure 21.** **Pumping thresholds of champion lasing materials.** Data is collected from literature reports (Supplementary Table 5 and 6) plotted as a function of emission wavelength (A) and aggregated by emitter type (B).


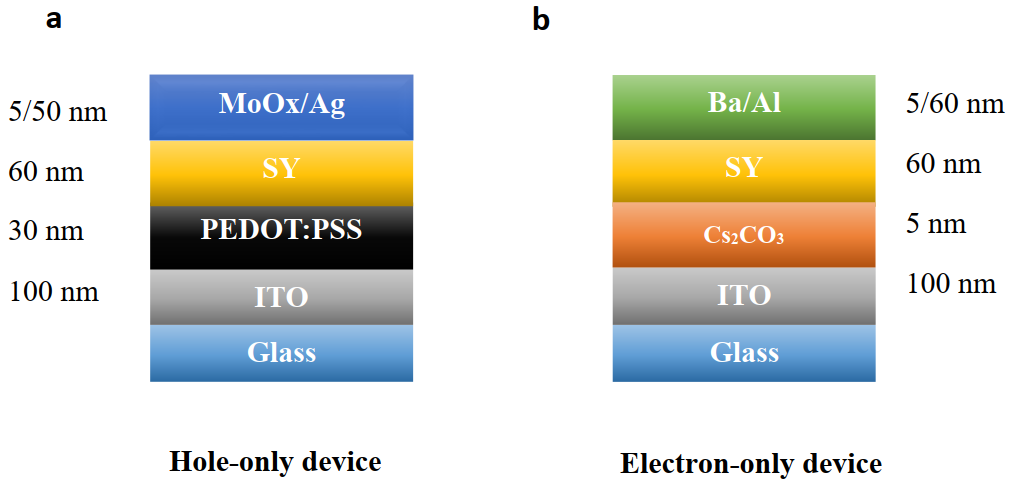


**Supplementary Figure 22. Structures of hole-only (HOD) and electron-only (EOD) devices. a** Device structures of hole-only (HOD). **b** electron-only device (EOD) for SY polymer with respective thicknesses.


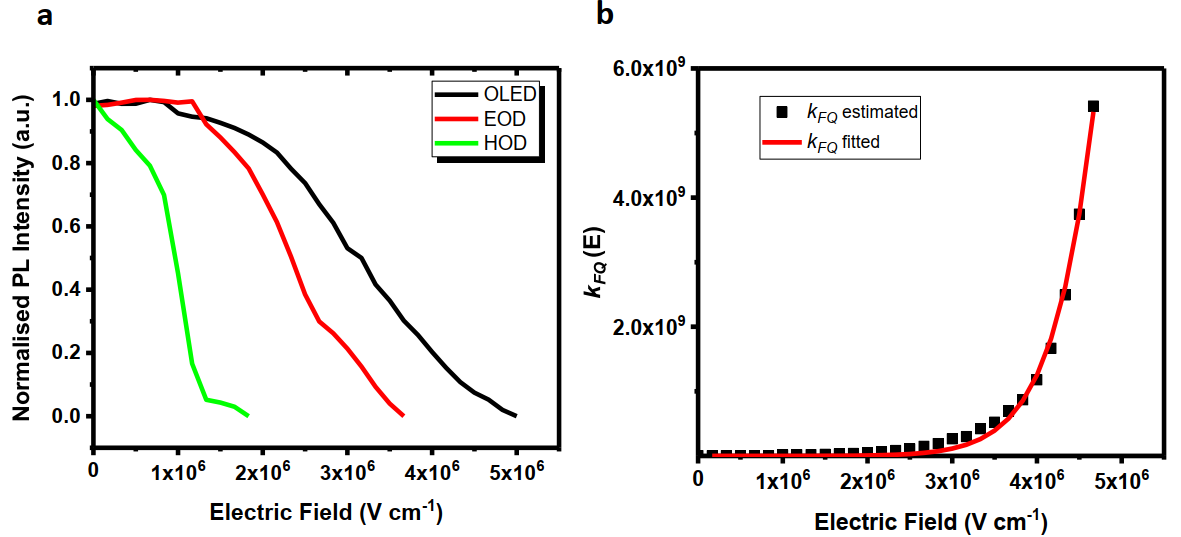


**Supplementary Figure 23.** **Results of field induced quenching PL study.** **a** Field induced quenching of PL intensity can be seen with increasing voltage/electric field. Field quenching results of OLED, hole-only and electron-only device are shown with hole-only device (HOD) being most affected. **b** Estimated *vs* fitted field quenching parameter (*k_FQ_*).


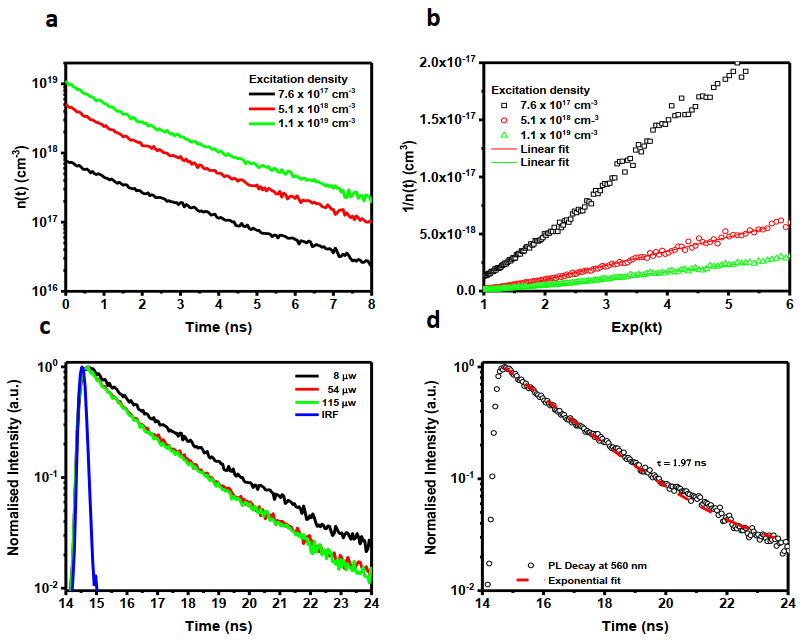


**Supplementary Figure 24.** **Measurement of Singlet-singlet annihilation (SSA) constant (*k_ss_*).** Intensity dependent photoluminescence study was used. **a** The excitation densities achieved at various powers. **b** Relation of inverse of excitation density to exponential term of **Supplementary Equation 22, 23**. The PL decay of SY thin films excited at different powers. **d** Linearized form of these decay curves were fit with equation S3c to obtain *k_ss_* from slope and the intercept of the linear fit. Summary of the results are shown in **Supplementary Table 7**.


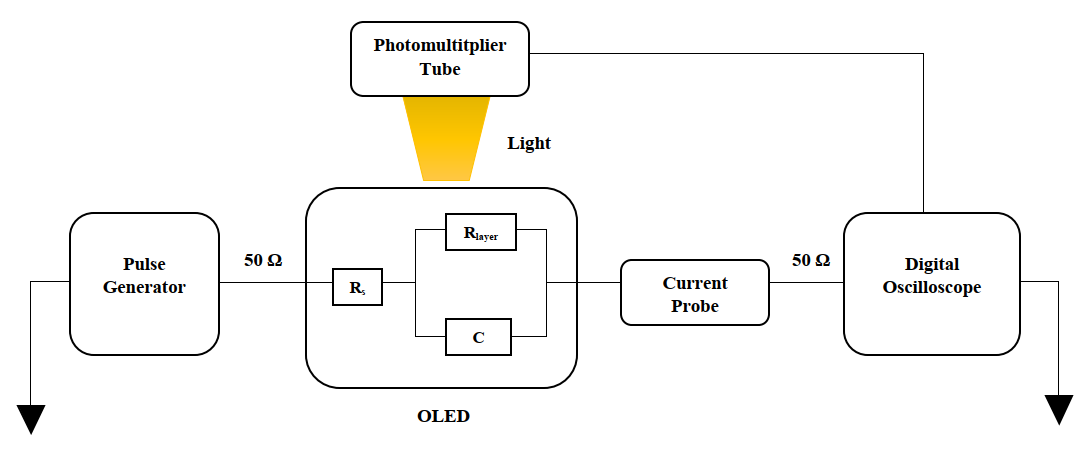


**Supplementary Figure 25. Schematic diagram of high voltage pulse measurement setup**. All instruments are terminated at 50 Ω resistance and cables used are of the same resistance to keep signal reflection negligible.

**Supplementary Table 1. Values of contact resistance R_s_, layer resistance R_layer_**, They are calculated from high voltage pulse inputs. These results combined with impedance spectroscopy measurement of capacitance helps in determining the RC/time constant of the device.

| Area (80 V input) | R_s_ (Ω) | R_layer_ (Ω) | Capacitance  (pF) | RC Constant  LCR (ns) | RC Constant Exp. Fit (ns) |
| --- | --- | --- | --- | --- | --- |
| 0.3 mm^2^ | (93 ± 5) | (368 ± 15) | (120 ± 10) | (8.0 ± 0.5) | (10 ± 1) |

**Supplementary Table 2.** **Decay and annihilation rates of F8BT** (theoretical/measured *vs* fitted)

| Decay Rates (s^-1^) | F8BT | Model |
| --- | --- | --- |
| Singlet Decay Rate (*k_S_*) | 1.1 × 10^9^ (ref. 6) | 4.5 × 10^8^ |
| Intersystem Crossing (*k_ISC_*) | 1.2 × 10^7^ (ref. 7) | 0.6 × 10^7^ |
| Triplet Decay Rate (*k_T_*) | – | 1 × 10^5^ |
| **Annihilation Rates (cm^3^ s^-1^)** |  |  |
| Singlet-Singlet (*k_SS_*) | 1.6 × 10^-9^ (ref. 6) | 8 × 10^-8^ |
| Singlet-Triplet (*k_ST_*) | 3 × 10^-13^ (ref. 8) | 9 × 10^-13^ |
| Triplet-Triplet (*k_TT_*) | 9 × 10^-12^ (ref. 9) | 9 × 10^-12^ |
| Singlet-Polaron (*k_SP_*) | 5 × 10^-14^ | 8 × 10^-14^ |
| Triplet-Polaron (*k_TP_*) | – | 4 × 10^-13^ |
|  | | |
| Exciton-Field Separation Constant (*k_FQ_*) | 850 s^-1^ (fitted) | 850 s^-1^ |
| Exciton-Field Separation Bessel Term (*k_FQ_*) | 2 × 10^8^ V m^-1^ (fitted) | 2 × 10^8^ V m^-1^ |
| Device Permittivity | 3.4 | |
| Active Layer Thickness | 70 nm | 70 nm |
| Hole Mobility | 1 × 10^-4^ (measured) | 1.992 × 10^-4^ |
| Electron Mobility | 3 × 10^-6^ (ref. 10) | 3.83 × 10^-5^ |

**Supplementary Table 3.** **Decay and annihilation rates of PFO (**theoretical/measured *vs* fitted)

| Decay Rates (s^-1^) | PFO | Model |
| --- | --- | --- |
| Singlet Decay Rate (*k_S_*) | 2.5 × 10^8^ (ref. 6) | 8 × 10^8^ |
| Intersystem Crossing (*k_ISC_*) | 5 × 10^7^ (ref. 11) | 5 × 10^7^ |
| Triplet Decay Rate (*k_T_*) | -- | 1 × 10^5^ |
| **Annihilation Rates (cm^3^ s^-1^)** |  |  |
| Singlet-Singlet (*k_SS_*) | 1.5 × 10^-8^ | 1.4 × 10^-8^ |
| Singlet-Triplet (*k_ST_*) | 5.2 × 10^-10^ (ref. 11) | 3.45 × 10^-11^ |
| Triplet-Triplet (*k_TT_*) | 1 × 10^-15^ (ref. 12) | 1 × 10^-15^ |
| Singlet-Polaron (*k_SP_*) | 5 × 10^-14^ | 5 × 10^-14^ |
| Triplet-Polaron (*k_TP_*) | -- | 4 × 10^-13^ |
|  | | |
| Exciton-Field Separation Constant (*k_FQ_*) | 850 s^-1^ (fitted) | 850 s^-1^ |
| Exciton-Field Separation Bessel Term (*k_FQ_*) | 2 × 10^8^ V m^-1^ (fitted) | 2 × 10^8^ V m^-1^ |
| Device Permittivity | 3.4 | |
| Active Layer Thickness | 70 nm | 70 nm |
| Hole Mobility | 2 × 10^-5^ | 2 × 10^-4^ |
| Electron Mobility | 2 × 10^-5^ (ref. 13) | 3.83 × 10^-5^ |

**Supplementary Table 4.** **Decay and annihilation rates of BSBCz** They were used to model the current threshold for the reported device.

| BSBCz Decay Rates (s^-1^) | Value obtained from literature (used as-is) |
| --- | --- |
| Singlet Decay Rate (*k_S_*) | 6 × 10^8^ (ref. 14) |
| Intersystem Crossing (*k_ISC_*) | 1 × 10^6^ (ref. 14) |
| Triplet Decay Rate (*k_T_*) | 1 × 10^6^ (ref. 14) |
| **Annihilation Rates (cm^3^ s^-1^)** |  |
| Singlet-Singlet (*k_SS_*) | 1 × 10^-7^ (ref. 15) |
| Singlet-Triplet (*k_ST_*) | 1 × 10^-12^ (ref. 15) |
| Triplet-Triplet (*k_TT_*) | – |
| Singlet-Polaron (*k_SP_*) | 1 × 10^-9^ (ref. 15) |
| Triplet-Polaron (*k_TP_*) | – |
| Exciton-Field Separation Constant (*k_FQ_*) |  |
| Exciton-Field Separation Bessel Term (*k_FQ_*) |  |
| Device Permittivity | 4 (ref. 14) |
| Active Layer Thickness | 200 nm (ref. 15) |
| Hole Mobility | 6 × 10^-4^ cm^2^ V^-1^ s^-1^ (ref. 14) |
| Electron Mobility | 2 × 10^-4^ cm^2^ V^-1^ s^-1^ (ref. 14) |
| Threshold generation term | 2.3 × 10^25^ cm^-3^ s^-1^ (ref.14) |

| Active Material | Excitation  (nm), (laser type) | ASE  (nm) | Threshold ASE | Threshold with cavity | Type of Cavity | Reference |
| --- | --- | --- | --- | --- | --- | --- |
| Small Molecules | | | | | | |
| difluorene with siloxane groups (monolithic liquid) | 337, (nitrogen) | 386 | 1.4 µJ cm^-2^ | – | – | ^18^ |
| C545T (1%) in mCBP co-doped with ACRXTN (6%) | 337, (nitrogen) | 535 | 0.8±0.3 µJ cm^-2^ | – | – | ^19^ |
| DABNA-2 | 337, (nitrogen) | 494 | 1.6±0.3 µJ cm^-2^ | – | – | ^20^ |
| BSBCz : CBP (6wt%) | 337, (nitrogen) | 461 | 0.32 µJ cm^-2^ | 0.090 µJ cm^-2^ | 140, 280 nm period (1-D, mixed order), | ^21^ |
| BSBCz-CN : CBP (6wt%) | 337, (nitrogen) | 512 | 0.63 µJ cm^-2^ | – | – | ^22^ |
| CzPV-SBF | 337, (nitrogen) | 474 | 0.4 µJ cm^-2^ | – | – | ^23^ |
| CzPV-SBF in CBP (6wt%) | 337, (nitrogen) | 474 | 0.11 µJ cm^-2^ | – | – | ^23^ |
| Oligomers | | | | | | |
| heptafluorene+CBP | 337, (nitrogen) | 446 | 0.32 µJ cm^-2^ | – | – | ^24^ |
| Octafluorene | 337, (nitrogen) | 450 nm | 0.090 µJ cm^-2^ | 0.084 nJ cm^-2^ | 260 and 130 nm (1-D, mixed order) | ^25^ |
| terfluorene | 325 | 423 | 1.3 µJ cm^-2^ | – | – | ^26^ |
| Pentafluorene | 325 | 442 | 1.2 µJ cm^-2^ | – | – | ^26^ |

**Supplementary Table 5. Best performing small molecules and oligomers.** These are literature reported materials showing low (E_th_ < 10 µJ cm^-2^) threshold for Amplified Spontaneous Emission or lasing.

| Active Material | Excitation  (nm), (laser type) | ASE  (nm) | Threshold ASE | Threshold with cavity | Type of Cavity | Reference |
| --- | --- | --- | --- | --- | --- | --- |
| Dendrimers | | | | | | |
| truxene star-shaped oligofluorenes | 380, (Nd3+:YAG) | 437 | – | 1.29 µJ cm^-2^ (Tr 6-3) | 260 nm period (1-D, second order), FF=75% | ^27^ |
| truxene star-shaped oligofluorenes | 380, (Nd3+:YAG) | 422-473 | 8.4 µJ cm^-2^ (Tr3-3) | 2.06 µJ cm^-2^ (Tr3-3) | 270 nm period (1-D, second order), FF=75% | ^28^ |
| truxene star-shaped oligofluorenes | 355, (Nd:YV04) | 428-453 | - | 1.1 µJ cm^-2^ (T4) | 270-301 nm period | ^29^ |
| Pyrene core with fluorene dendrons as antenna | 380, (Nd3+:YAG) | 491 | 28, 25 nJ pulse^-1^ | 0.15, 0.26 µJ cm^-2^ | 320 nm period (1-D), FF=75% | ^30^ |
| Polymers | | | | | | |
| VE-PFO (B-phase) | 355, (Nd3+:YAG) | 467 | – | 0.4 µJ cm^-2^ | 254 nm period (1-D, second order) | ^31^ |
| PFO:PFO-EH copolymer (4:1) | 390 | 447 | – | 0.3 µJ cm^-2^ | 290 nm period (1-D), FF=75% | ^32^ |
| terphenylenevinylene polymer (BBEHP-PPV) | 337, (nitrogen) | 535 | 0.33 µJ cm^-2^ | 0.04 µJ cm^-2^ | Not mentioned | ^33^ |
| DCz-LPh5 | 375 nm, Nd3+:YAG | 447 nm | 10±2 nJ pulse^-1^ | 0.18 µJ cm^-2^ | 290 nm period (1-D , second order), FF=50% | ^34^ |
| F8DP | 390 nm, Nd3+:YAG | 452 nm | - | 0.036 µJ cm^-2^ | 140 and 280 nm(mixed order) | ^35^ |
| CPDHFPV | 337 nm, nitrogen | 513 nm | 0.16 µJ cm^-2^ | - | - | ^36^ |
| CPDHFPV in PVK (5%) | 337 nm, nitrogen | 514 nm | 0.02 µJ cm^-2^ | - | - | ^36^ |

**Supplementary Table 6. Best performing dendrimers and polymers.** These literatures reported materials show low (E_th_ < 10 µJ cm^-2^) threshold for Amplified Spontaneous Emission or lasing.

**Supplementary Table 7. Estimation of singlet-singlet annihilation rate**

| **Power (µW)** | **Excitation density (cm^-3^)** | ***k_ss_* (slope) (×10^-10^)** | ***k_ss_* (intercept) (×10^-10^)** |
| --- | --- | --- | --- |
| 54 | 5.1 × 10^18^ | 1.5 | 2.2 |
| 115 | 1.1 × 10^19^ | 3.2 | 4.5 |
| Average | | 2.9 ± 1.3 | |

**Supplementary Note 1. Circuit model – square pulse response**

Solution to the system of equations describing the circuit (**Equation 1**, **2**), driven by square excitation pulse of magnitude *V*_0_*_,_* is given by **Supplementary Equation 1**, **2** for the pulse duration and after the pulse ends, respectively, with *τ* given by **Supplementary Equation 3,** being the characteristic operation time of the device.

$I_{\mathrm{on}}(t)=\frac{V_{0}}{R_{s}}\left( \frac{R_{s}}{R_{s}+R_{\mathrm{layer}}}-\left( \frac{R_{s}}{R_{s}+R_{\mathrm{layer}}}-1 \right)e^{-t/\tau} \right)$ (1)

$I_{\mathrm{off}}\left( t \right)=-\frac{V_{0}}{R_{s}}\left( \frac{R_{\mathrm{layer}}}{R_{s}+R_{\mathrm{layer}}}e^{-t/\tau} \right)$ (2)

$\tau=C\frac{R_{s}{╳ R}_{\mathrm{layer}}}{R_{s}+ R_{\mathrm{layer}}}$ (3)
 From the analytical solution, one can see that the peak current (*t* = 0) is equal to *V*_0_*/R*_s_ and is independent of the organic layer. Steady state current, on the other hand, is the result of organic layer reaching equilibrium state at a given potential and is equal to *V*_0_*/(R*_s_*+R*_laye_*_r_)*.

**Supplementary Note 2. Circuit model – trapezoid pulse response**

In the trapezoid excitation case, the characteristic time of the device remains the same and current solution to **Equation 1**, **2** is given by **Supplementary Equation 4-7.**

$I_{\mathrm{rise}}(t)=\frac{V_{0}}{R_{s}}\tau\frac{1}{t_{\mathrm{rise}}}\left[ \left( 1-e^{-t/\tau} \right)\frac{R_{\mathrm{layer}}}{R_{s}+R_{\mathrm{layer}}}+\frac{t}{CR_{\mathrm{layer}}} \right]$ (4)

$I_{\mathrm{flat}}(t)=\frac{V_{0}}{R_{s}}\left( \frac{R_{s}}{R_{s}+R_{\mathrm{layer}}}-\left( \frac{R_{s}}{R_{s}+R_{\mathrm{layer}}}-1 \right)\left( 1-n \right)e^{-t/\tau} \right)$ (5)

$I_{\mathrm{fall}}\left( t \right)=-\frac{V_{0}}{R_{s}}\frac{1}{t_{\mathrm{fall}}}\left[ \tau\left( 1-\frac{R_{s}}{R_{s}+R_{\mathrm{layer}}} \right)-\left( \frac{R_{s}}{R_{s}+R_{\mathrm{layer}}} \right)\left( t_{\mathrm{fall}}-t \right)-\left( 1- \frac{R_{s}}{R_{s}+R_{\mathrm{layer}}} \right)\left( \tau+t_{\mathrm{fall}}\left( 1-n \right) \right)e^{-t/\tau} \right]$  (6)

$I_{\mathrm{off}}=-\frac{V_{0}}{R_{s}}n\left( \frac{R_{\mathrm{layer}}}{R_{s}+R_{\mathrm{layer}}} \right)e^{-t/\tau}$ (7)

where *I_rise_, I_flat_* and *I_fall_* represent rise, flat and fall current in response to trapezoidal input pulse and *t_fall_* is the fall time of the voltage pulse. One can see that the steady-state current value obtained for the equilibrium state will be the same as in the square pulse case, as expected. The height of the initial peak, however, will depend on the parameter *n* (taking values from 0 to 1) which describes the level of charge stored in the capacitor during the previous step as a fraction of its maximum value of $\frac{V_{0}}{R_{s}}\tau$ (n = 1).

**Supplementary Note 3. Drift-diffusion model**

The drift diffusion model of the OLEDs organic layer is given by **Supplementary** **Equation 8-18** below:

$j_{p}\left( x,t \right)=e\mu_{p}E\left( x,t \right)p\left( x,t \right)-\mu_{p}k_{B}T\frac{\partial p}{\partial x}$ (8)
 $j_{n}\left( x,t \right)=e\mu_{n}E\left( x,t \right)n\left( x,t \right)+\mu_{n}k_{B}T\frac{\partial n}{\partial x}$ (9)
 $\frac{\partial p}{\partial t}+\frac{1}{e}\frac{\partial j_{p}}{\partial x}=-\beta np$ (10)
$\frac{\partial n}{\partial t}-\frac{1}{e}\frac{\partial j_{n}}{\partial x}=-\beta np$ (11)
$\frac{\partial^{2}V}{\partial x^{2}}=\frac{n-p}{\epsilon\epsilon_{0}}$ (12)
$E=-\frac{\partial V}{\partial x}$ (13)
$V_{c}\left( t \right)=\int_{0}^{d} E\left( x,t \right)dx$ (14)

$i_{c}=\frac{S}{d} \int_{0}^{d} j_{p}\left( x \right)+j_{n}\left( x \right)dx$ (15)
$R_{s}C\frac{dV_{c}}{dt}=V_{\mathrm{in}}\left( t \right)-V_{c}\left( t \right)-i_{c}R_{s}$ (16)

$\mu_{n}\left( t \right)=\mu_{0,n}\left( (1-\mu_{SS,n})exp \left( -\frac{t}{\tau} \right)+\mu_{\mathrm{SS},n} \right)$ (17)

$\mu_{p}\left( t \right)=\mu_{0,p}\left( (1-\mu_{\mathrm{SS},p})exp \left( -\frac{t}{\tau} \right)+\mu_{\mathrm{SS},p} \right)$ (18)

Here, **Supplementary Equation 8**, **9** describe the current density in the system with *j_p_*(*j_n_*) being hole(electron) current density, *p*(*n*) being hole(electron) density, *μ*_p_(*μ*_n_) being hole(electron) mobility, *k_B_* being Boltzmann constant, *T* being temperature, *ε_0_* as the permittivity of free space, *ε_r_* is the relative permittivity of the emission layer and $\beta$ being the bimolecular recombination coefficient. These equations have a drift and diffusive component, with the latter being expressed in terms of mobility *via* Einstein relation. Next, **Supplementary Equation 10**, **11** are charge continuity equations for holes and electrons, respectively. The right hand sides of **Supplementary Equation 10**, **11** describe bimolecular recombination of electrons and holes, where *β* is the Langevin recombination constant, since Langevin recombination is common in many organic systems^1^. Then, **Supplementary Equation 12, 13** are used to obtain electric field distribution in the organic layer, with **Supplementary Equation 14** putting the boundary conditions and *U(t)* being the total potential drop across the organic layer (with d being its thickness). Following that, **Supplementary Equation 15** defines the current flowing through the organic layer, with *S* being the cross-sectional area of the device stack. External circuitry is incorporated into the model through **Supplementary Equation 16** in order to account for RC circuit dynamics, with $V_{c}\left( t \right)$ being the voltage across the semiconductor and $V_{\mathrm{in}}\left( t \right)$ the power supply voltage. Finally, trapping was implemented through the inclusion of a time dependent device averaged mobility given in **Supplementary Equation 17**, **18**, where it has previously been shown that certain types of trapping exhibit an exponential decay ^2^, with $\mu_{0,p}$($\mu_{0,n})$ being the initial mobility of holes(electrons) and $\mu_{SS,p}$($\mu_{SS,n})$ the steady-state mobility of holes(electrons).

For initial fits, our model employs Boltzmann Ohmic boundary conditions, wherein the injecting and extracting charge carrier densities are fixed ^3^. While this allows for accurate modelling of experimental behaviour, in order to ensure Ohmic injection for lasing threshold simulations, which sample a large range of applied voltages, it was necessary to employ ideal injecting boundary conditions, meaning that we neglect any contact limitations and assume that the injection current is always space charge limited.

This is a justifiable assumption for high current OLEDs when driven with large applied voltages. The numerical implementation of this ideal Ohmic injection is to calculate the charge densities at the injecting interfaces in order to drive the electric field at that interface to near zero ^4^. The boundary condition at the extracting interface is given by the drift current outflow ($j_{p}=e\mu_{p}Ep$ and similarly for electrons), however, the results are insensitive to the extracting electrode’s boundary condition because the vast majority of charges do not actually reach the opposite electrode in a film with Langevin recombination. Furthermore, many optimised OLED devices include blocking layers to guarantee that charges are not able to escape the emissive layer.

Drift-diffusion models such as this make several simplifying assumptions that should be considered. The model is one dimensional, and so necessarily must average over any lateral variations in film properties and any lateral motion of charge carriers. Trapping is considered only through its effect on mobility, i.e. the model does not consider the microscopic process of charge trapping. It is possible to extend the model by introducing additional continuity equations for trapped electrons and trapped holes, and then specifying rate constants for charges to enter and leave the trap sites. However, this adds many new degrees of freedom to the model, and so instead, we average out the effect of trapping and consider the resulting effective mobility that results from a mix of trapped and untrapped charges. Furthermore, the model assumes a homogeneous effective medium for the emissive layer. It would be possible to extend the model to include spatial variation in the parameters if necessary. Overall, despite these simplifying assumptions, drift-diffusion models are known to strike a good balance between interpretability and accuracy^5^.

**Supplementary Note 4. Hole and electron-only devices and field quenching experiments**

The device structure and thickness employed for hole and electron-only devices are shown in **Supplementary Figure 22**. The same procedures for fabrication and measurement were followed as mentioned in experimental section.

**Thickness and dielectric constant measurements**

The thickness of SY polymer was found by first spin coating 7 mg mL^-1^ solution of SY (in toluene) on a clean quartz substrate and then measuring the thickness using a Dektak 150 profilometer. The dielectric constant of the polymer was calculated from impedance spectroscopy measurements. First, capacitance was measured for a set thickness and area of device. The measured capacitance was then used to find dielectric constant using **Supplementary Equation 19** below:

$C= \frac{Ɛ_{0}Ɛ_{r} A}{d}$(19)

where *C* is the measured capacitance of the organic layer, $Ɛ_{0}$is the permittivity of air, $Ɛ_{r}$ is the unknown dielectric constant, *A* and *d* are area and thickness of the organic layer, respectively.

**Field quenching experiment details**

Hole and electron only-devices (with details above) were subjected to DC voltage from 0 to 30 V in steps of 1 V with an incident nitrogen laser to determine quenching effects. Resulting PL intensities, as shown in **Supplementary Figure 21a**, were recorded to determine exciton binding energy of SY which was then used to determine field quenching parameter (*k_FQ_*), by a method reported earlier using the **Supplementary Equation 20**^16^

$$k_{FQ}= \frac{3\gamma}{4\pi r^{3}}\exp( \frac{{-E}_{b}}{k_{B}T})\frac{J_{1}(2\sqrt{\left. -2b \right)}}{\sqrt{-2b}} (20)$$

where $\gamma$ is the mobility-dependent Langevin recombination factor, *r*  is electron-hole radius, *E_b_* is exciton binding energy, *k_B_T* is thermal energy, *J_1_* is Bessel function of first order and $b=e^{3}F/(8\piƐ_{o}Ɛ_{r}(k_{B}T)^{2})$ (e is elementary charge, F is electric field and $Ɛ_{o}Ɛ_{r}$ is effective dielectric constant).

**Supplementary Note 5. Measurement of singlet-singlet annihilation (SSA) constant (*k_ss_*)**

Singlet-Singlet annihilation rate constant (*k*_ss_) for super yellow thin films was determined by technique as shown in literature^17^. Thin films (≈120 nm) were excited at 450 nm using optical parametric amplifier with output from amplified laser system (spitfire ACE, spectra physics) delivering ≈100 fs laser pulses at 800 nm operating at 1 kHz as the feed. Photoluminescence (PL) decay was measured using Halcyone fluorescence spectrometer at different excitation intensities from 8–115 µW (area ≈0.002 cm^2^) which was controlled using neutral density filters. The PL decay from the thin films can be correlated to *k*_ss_ using the following equation:

$\frac{dn\left( t \right)}{dt}=-kn\left( t \right)-k_{\mathrm{ss}}n^{2}(t)$ (21)

where *n(t)* are the number of carriers*.* Above equation can be solved to obtain the following:

$n(t)=\frac{n\left( 0 \right)e^{-kt}}{1+\left( \frac{k_{\mathrm{ss}}}{k} \right)n\left( 0 \right)\left[ 1-e^{-kt} \right]}$ (22)

$\frac{1}{n\left( t \right)}=\left( \frac{1}{n\left( 0 \right)}+\frac{k_{\mathrm{ss}}}{k} \right)e^{kt}-\frac{k_{\mathrm{ss}}}{k}$ (23)

**Supplementary Note 6.** Light Outcoupling

In order to be able to fit the experimental brightness transients, our model has a parameter linking the number of singlets undergoing radiative decay and the device brightness. The value of this parameter is obtained using the Setfos package to simulate our device stack based on complex refractive indices and PL spectra of the materials and device architecture. As can be seen in **Supplementary Figure 20**, keeping the same device stack for different emitters results in fairly consistent outcoupling channels. We also included outcoupling results for the device reported by Adachi’s group, which we simulated in the manuscript.

**Supplementary References**

1. Van Der Holst, J. J. M., Van Oost, F. W. A., Coehoorn, R. & Bobbert, P. A. Electron-hole recombination in disordered organic semiconductors: Validity of the Langevin formula. *Phys. Rev. B - Condens. Matter Mater. Phys.* **80**, 235202 (2009).

2. Stokes, P. W., Philippa, B., Cocks, D. & White, R. D. Solution of a generalized Boltzmann’s equation for nonequilibrium charged-particle transport via localized and delocalized states. *Phys. Rev. E* **93**, 032119 (2016).

3. Koster, L. J. A., Smits, E. C. P., Mihailetchi, V. D. & Blom, P. W. M. Device model for the operation of polymer/fullerene bulk heterojunction solar cells. *Phys. Rev. B - Condens. Matter Mater. Phys.* **72**, 085205 (2005).

4. Lampert, M. A. & Schilling, R. B. Chapter 1 Current Injection in Solids: The Regional Approximation Method. in *Injection Phenomena* (eds. Willardson, R. K. & Beer, A. C. B. T.-S. and S.) **6**, 1–96 (Elsevier, 1970).

5. Heiber, M. C., Wagenpfahl, A. & Deibel, C. 10 - Advances in modeling the physics of disordered organic electronic devices. in *Woodhead Publishing Series in Electronic and Optical Materials* (ed. Ostroverkhova, O. B. T.-H. of O. M. for E. and P. D. (Second E.) 309–347 (Woodhead Publishing, 2019).

6. Shaw, P. E. Measurements of exciton diffusion in conjugated polymers. (University of St. Andrews, 2009).

7. Ompong, D. & Singh, J. Study of intersystem crossing mechanism in organic materials. *Phys. Status Solidi Curr. Top. Solid State Phys.* (2016).

8. Dey, A., Rao, A. & Kabra, D. A Complete Quantitative Analysis of Spatio-Temporal Dynamics of Excitons in Functional Organic Light-Emitting Diodes. *Adv. Opt. Mater.* **5**, 1600678-n/a (2017).

9. Lehnhardt, M., Riedl, T., Rabe, T. & Kowalsky, W. Room temperature lifetime of triplet excitons in fluorescent host/guest systems. *Org. Electron.* **12**, 486–491 (2011).

10. Zhang, Y. & Blom, P. W. M. Electron and hole transport in poly(fluorene-benzothiadiazole). *Appl. Phys. Lett.* **98**, 143504 (2011).

11. Gärtner, C., Karnutsch, C., Lemmer, U. & Pflumm, C. The influence of annihilation processes on the threshold current density of organic laser diodes. *J. Appl. Phys.* **101**, 23107 (2007).

12. Scherf, U. & Neher, D. *Polyfluorenes*. (Springer Berlin Heidelberg, 2008).

13. Nicolai, H. T. *et al.* Space-charge-limited hole current in poly(9,9-dioctylfluorene) diodes. *Appl. Phys. Lett.* (2010).

14. Sandanayaka, A. S. D. *et al.* Indication of current-injection lasing from an organic semiconductor. *Appl. Phys. Express* **12**, 61010 (2019).

15. Bencheikh, F., Sandanayaka, A. S. D., Fukunaga, T., Matsushima, T. & Adachi, C. Origin of external quantum efficiency roll-off in 4,4′-bis[(N-carbazole)styryl]biphenyl (BSBCz)-based inverted organic light emitting diode under high pulsed electrical excitation. *J. Appl. Phys.* **126**, 185501 (2019).

16. Kern, J., Schwab, S., Deibel, C. & Dyakonov, V. Binding energy of singlet excitons and charge transfer complexes in MDMO-PPV: PCBM solar cells. *Phys. Status Solidi - Rapid Res. Lett.* **5**, 364–366 (2011).

17. Shaw, P. E., Ruseckas, A. & Samuel, I. D. W. Exciton Diffusion Measurements in Poly(3-hexylthiophene). *Adv. Mater.* **20**, 3516–3520 (2008).

18. Ribierre, J.-C. *et al.* Low threshold amplified spontaneous emission and ambipolar charge transport in non-volatile liquid fluorene derivatives. *Chem. Commun. (Camb).* **52**, 3103–3106 (2016).

19. Nakanotani, H., Furukawa, T. & Adachi, C. Light amplification in an organic solid-state film with the aid of triplet-to-singlet upconversion. *Adv. Opt. Mater.* **3**, 1381–1388 (2015).

20. Nakanotani, H., Furukawa, T., Hosokai, T., Hatakeyama, T. & Adachi, C. Light Amplification in Molecules Exhibiting Thermally Activated Delayed Fluorescence. *Adv. Opt. Mater.* **5**, 1700051 (2017).

21. Sandanayaka, A. S. D. *et al.* Toward continuous-wave operation of organic semiconductor lasers. *Sci. Adv.* **3**, e1602570 (2017).

22. Mamada, M., Fukunaga, T., Bencheikh, F., Sandanayaka, A. S. D. & Adachi, C. Low Amplified Spontaneous Emission Threshold from Organic Dyes Based on Bis-stilbene. *Adv. Funct. Mater.* **28**, 1802130 (2018).

23. Nakanotani, H. *et al.* Extremely Low-Threshold Amplified Spontaneous Emission of 9,9′-Spirobifluorene Derivatives and Electroluminescence from Field-Effect Transistor Structure. *Adv. Funct. Mater.* **17**, 2328–2335 (2007).

24. Zhao, L. *et al.* Singlet-Triplet Exciton Annihilation Nearly Suppressed in Organic Semiconductor Laser Materials Using Oxygen as a Triplet Quencher. *IEEE J. Sel. Top. Quantum Electron.* **22**, 26–34 (2016).

25. Kim, D.-H. *et al.* Extremely low amplified spontaneous emission threshold and blue electroluminescence from a spin-coated octafluorene neat film. *Appl. Phys. Lett.* **110**, 23303 (2017).

26. Choi, E. Y. *et al.* Photophysical, amplified spontaneous emission and charge transport properties of oligofluorene derivatives in thin films. *Phys. Chem. Chem. Phys.* **16**, 16941–16956 (2014).

27. Lai, W.-Y. *et al.* Enhanced Solid-State Luminescence and Low-Threshold Lasing from Starburst Macromolecular Materials. *Adv. Mater.* **21**, 355–360 (2009).

28. Wang, Y. *et al.* Broadly tunable deep blue laser based on a star-shaped oligofluorene truxene. *Synth. Met.* **160**, 1397–1400 (2010).

29. Tsiminis, G. *et al.* Low-threshold organic laser based on an oligofluorene truxene with low optical losses. *Appl. Phys. Lett.* **94**, 243304 (2009).

30. Xia, R., Lai, W.-Y., Levermore, P. A., Huang, W. & Bradley, D. D. C. Low-Threshold Distributed-Feedback Lasers Based on Pyrene-Cored Starburst Molecules with 1,3,6,8-Attached Oligo(9,9-Dialkylfluorene) Arms. *Adv. Funct. Mater.* **19**, 2844–2850 (2009).

31. Kuehne, A. J. C. *et al.* Sub-Micrometer Patterning of Amorphous- and β-Phase in a Crosslinkable Poly(9,9-dioctylfluorene): Dual-Wavelength Lasing from a Mixed-Morphology Device. *Adv. Funct. Mater.* **21**, 2564–2570 (2011).

32. Yap, B. K., Xia, R., Campoy-Quiles, M., Stavrinou, P. N. & Bradley, D. D. C. Simultaneous optimization of charge-carrier mobility and optical gain in semiconducting polymer films. *Nat. Mater.* **7**, 376–380 (2008).

33. Rose, A., Zhu, Z., Madigan, C. F., Swager, T. M. & Bulović, V. Sensitivity gains in chemosensing by lasing action in organic polymers. *Nature* **434**, 876–879 (2005).

34. Wei, Q. *et al.* A High Performance Deep Blue Organic Laser Gain Material. *Adv. Opt. Mater.* **5**, 1601003 (2017).

35. Karnutsch, C. *et al.* Improved organic semiconductor lasers based on a mixed-order distributed feedback resonator design. *Appl. Phys. Lett.* **90**, 131104 (2007).

36. Lee, T.-W., Park, O. O., Choi, D. H., Cho, H. N. & Kim, Y. C. Low-threshold blue amplified spontaneous emission in a statistical copolymer and its blend. *Appl. Phys. Lett.* **81**, 424–426 (2002).
